# Internal Fixation of Intertrochanteric Hip Fractures: A Clinical Comparison of Two Implant Designs

**DOI:** 10.1155/2013/834825

**Published:** 2013-02-17

**Authors:** Ran Tao, Yue Lu, Hua Xu, Zhen-Yu Zhou, You-Hua Wang, Fan Liu

**Affiliations:** Department of Orthopaedics, Affiliated Hospital of Nantong University, 20 Xisi Road, Nantong, Jiangsu Province 226001, China

## Abstract

*Objective*. To compare two internal fixation devices clinically in stabilisation of intertrochanteric femur fractures. *Methods*. Eighty-seven patients were randomised upon their admission to the hospital using a sealed envelope method. Forty-five were treated with proximal femur nail antirotation (PFNA) and 42 with reverse less invasive stabilisation system (LISS). The perioperative data were recorded and compared in relation to fracture type. *Results*. In each type of fractures, no significant differences were found with respect to the blood loss, the quality of reduction, the time to bony healing, and the Harris hip score between the 2 groups. The mean duration of surgery was significantly longer in reverse LISS group than in PFNA group. *Conclusion*. Both the PFNA and the reversed LISS are effective in the treatment of different types of intertrochanteric femur fractures. PFNA is superior to reverse LISS in terms of surgical time, weight-bearing, and perhaps fluoroscopy time.

## 1. Introduction

Numerous internal fixation devices have been used to stabilise intertrochanteric femur fractures, and they can be divided into 2 categories: extramedullary fixation devices and intramedullary fixation devices. It is generally accepted that dynamic hip screw (DHS) is the implant of choice in the treatment of stable intertrochanteric femur fractures (AO/OTA 31-A1) [1]. For unstable intertrochanteric femur fractures (AO/OTA 31-A2 and 31-A3), the commonly used extramedullary fixation devices, such as DHS, dynamic condylar screw (DCS), and angular blade plates are sometimes problematic [1–3]. The less invasive stabilisation system-distal femur (LISS-DF) was designed for stabilisation of distal femur fracture. Recently, quite a few reports have addressed its use in the treatment of proximal femur fracture, [2, 4, 5] and the clinical results are encouraging. As to intramedullary fixation devices, so far, proximal femur nail antirotation (PFNA) is one of the most effective methods in the treatment of intertrochanteric femur fractures [6–10]. The purpose of the present study was to compare reverse LISS with PFNA clinically and to determine the preferred method in stabilisation of intertrochanteric femur fractures.

## 2. Patients and Methods

Between September 2010 and August 2011, 100 patients with 100 intertrochanteric femoral fractures were randomised upon their admission to the hospital using a sealed envelope method. The inclusion criteria were ages above 65. Those with pathological fractures, osteoarthritis of the hips, and ASA [11] (American Society of Anesthesiologists scale) 4 or 5 were excluded from the study. Of the 100 patients, 7 died of different causes unrelated to implants within 1 year and six was lost to followup. The remaining 87 patients were available for analysis. There were 33 men and 54 women. Forty-five of them were treated with PFNA (group I) and 42 with reverse LISS (group II). The fractures were classified according to AO/OTA classification. GroupI consisted of 10 cases of type 31A1, 21 cases of 31A2 and 14 cases of 31A3 fractures and group II, 9 cases of type 31A1, 21 cases of 31A2, and 12 cases of 31A3 fractures. The perioperative data, such as operative time, the overall fluoroscopy time, and the blood loss were noted and compared among the groups (Table 1). Ethical approval was obtained from the hospital.

**Figure 1:**
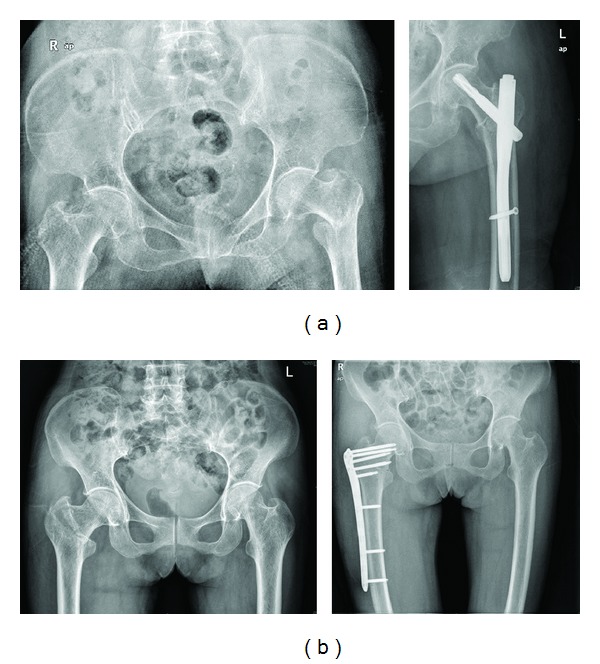
Patients with 31 A1 fractures. (a) PFNA: preoperative AP view and immediate postoperative AP view. (b) Reverse LISS: preoperative AP view and immediate postoperative AP view.

**Figure 2:**
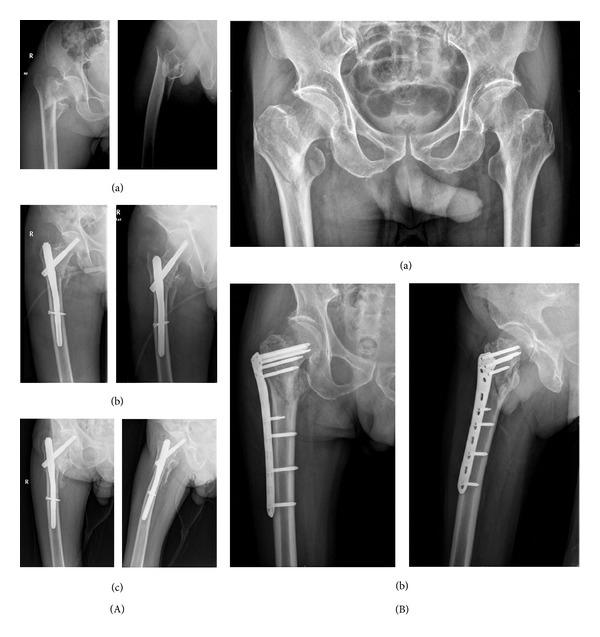
Patients with 31 A2 fractures. (A) PFNA (A(a)) Preoperative AP view and lateral view. (A(b)) Immediate postoperative AP view and oblique view. (A(c)) Three months postoperatively. Callus formation can be seen in both AP view and oblique view. (B) Reverse LISS (B(a)) Preoperative AP view. (B(b)) Immediate postoperative AP view and lateral view.

**Figure 3:**
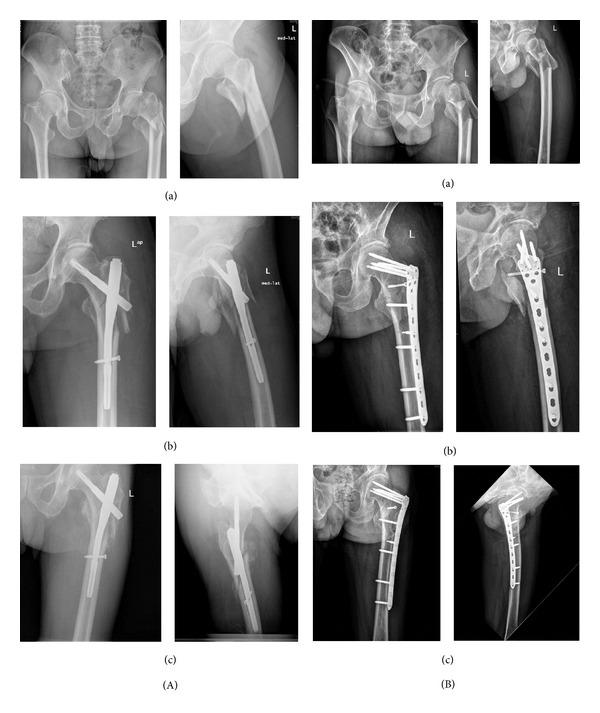
Patients with 31 A3 fractures. (A) PFNA (A(a)) Preoperative AP view and lateral view. (A(b)) Immediate postoperative AP view and lateral view. (A(c)) Six weeks postoperatively. Callus formation can be seen in both AP view and oblique view. (B) Reverse LISS (B(a)) Preoperative AP view and lateral view. (B(b)) Immediate postoperative AP view and lateral view. (B(c)) Six months postoperatively. Reverse LISS successfully maintained the reduction till bony healing.

All patients were evaluated preoperatively with use of two standard plain radiographs, an anterior-posterior radiograph, and a medial-lateral radiograph. Surgical treatment was performed as soon as the patient's general medical condition allowed. Prophylactic intravenous first generation cephalosporin was administered half an hour before operation and discontinued 48–72 hours postoperatively. Internal fixation was performed by 3 orthopaedic consultants (Figures 1, 2, and 3). Close reduction was carried out first in all the cases with patient supine on a traction table. If failed, then semiopen or open reduction was performed. Reverse LISS was used in a similar way described by Ma H et al. [2]. The quality of reduction was graded as good, acceptable (5–10° varus/valgus and/or anteversion/retroversion), or poor (>10° varus/valgus and/or anteversion/retroversion) [12]. Fractures were judged to be healed radiographically if bridging callus was evident on three of four cortices as seen on two views [13]. Intraoperative time was recorded from the time that the close reduction was started to the time that the wound was sutured closed.

In group I, partial and full weight-bearing were allowed on third and sixth postoperative week, respectively. In group II, these were postponed to 6th and 12th postoperative week, respectively. A follow-up evaluation, which included a clinical and radiographic assessment, was performed at 6, 13, 26, and 52 weeks. Functional outcomes were assessed according to the Harris hip scoring system [14]. 

Statistical analysis was performed with SPSS Statistics 11.5, with use of the Student's *t*-test, the chi square test. Statististical significance was defined as *P* < 0.05.

## 3. Results

The results in relation to treatment group are shown in Table 1.

In each type of fractures, no significant differences were found with respect to the age, the sex, the time from injury to surgery, the quality of reduction, the blood loss, the time to bony healing, and the Harris hip score between the 2 groups. The mean duration of surgery was significantly longer in group II than in groupI.

In type 31 A1 fractures, both the time to begin with partial weight-bearing (*P* < 0.001) and full weight-bearing (*P* < 0.001) were significant earlier in group I than in group II. The fluoroscopy time was significantly longer in group II than in group I. No significant difference was found with respect to the time of hospital stay.

In type 31 A2 fractures, no significant differences were found with respect to the fluoroscopy time and the time of hospital stay.

In type 31 A3 fractures, the patients in group I had significantly shorter time of hospital stay than in group II. No significant differences were found with respect to the fluoroscopy time.

In both 31 A2 and 31 A3 fractures, no significant differences were found regarding to the time to bony healing between the 2 groups. For most cases in group I, partial weight-bearing and full weight-bearing were began at 3 weeks and 6 weeks postoperatively, while in group II, most patients were allowed to start partial and full weight-bearing on 6th and 12th postoperative week, respectively.

Regardless of fracture types, no significant differences were found with respect to the age, the sex, the time from injury to surgery, the quality of reduction, the blood loss, the time of hospital stay, and the Harris hip score between the 2 groups. The patients in group I had significantly shorter duration of surgery, less fluoroscopy time as well as less time to obtain bone healing compared with that of in group II.

There were altogether 9 postoperative complications, including 3 cases of pressure sore, 3 cases of urinary infection, 2 cases of pulmonary infection, and 1 case of deep venous thrombosis. Loss of reduction, implant failure, and nonunion were not found in any case.

## 4. Discussion

Controversy persists concerning the optimal internal fixation devices for stabilisation of intertrochanteric femur fractures. Recently, there is a tendency of increased use of intramedullary nails [15, 16]. Theoretically, intramedullary fixation offers advantages over plates, especially in its ability to ensure stability even in unstable fractures. This was confirmed by the meta-analysis by Zeng et al. [17], who compared PFNA with DHS. However, the meta-analysis by Parker and Handoll of all prospective randomised trials comparing intramedullary to extramedullary devices does not support the perceived superiority of nails [1]. The purpose of the present study was to compare reverse LISS with PFNA in stabilisation of intertrochanteric femur fractures. To our knowledge, few authors [18, 19] compared these 2 devices clinically, and no published literatures made the comparison in relation to the fracture type.

The study population and the baseline data (age, sex, preoperative walking ability, and the duration from injury to surgery) were similar in each fracture type between the 2 groups. The most important finding of this study was that PFNA could significantly shorten surgical time compared with reverse LISS (31A1, *P* < 0.001; 31A2, *P* < 0.001; 31A3, *P* = 0.001; overall, *P* < 0.001). PFNA also shortened fluoroscopy time, but not statistically significant in unstable fractures (31A2 and 31A3). This can be explained that we are very familiar with PFNA [10] and lack of experience in reverse LISS. Before this study, only 4 intertrochanteric femur fractures (1 adolescent fracture, 3 pathological fractures) were treated by the contralateral reverse LISS-DF in our department. We found the correct positioning of reverse LISS to proximal femur was sometimes time consuming. There is no a so-called standard position concerning how proximal of the proximal end of LISS should be placed; however, two issues should be guaranteed. Firstly, at least 4 locking screws should be inserted in the proximal end of the LISS to effectively stabilise proximal fragment. Secondly, the LISS should be placed on the exact lateral aspect of the femur. PFNA shortened surgical time but did not reduce blood loss.

Good results were achieved with both the reverse LISS and PFNA in each fracture type, which was in accordance with the findings by Zhou et al. [19] and Han et al. [18]. Harris hip scores were comparable in both groups in relation to each fracture type. Another important finding of this study was that not a single mechanical failure was found in all the 87 fractures. This probably contributed to good quality of reduction, properly positioning of the internal fixation devices, as well as more conservative rehabilitation program. Every effort was made to obtain best reduction and ideal implants positioning. On rare occasions, close reduction was not satisfactory and open reduction was performed (3 cases in PFNA group, 4 cases in LISS group). As to postoperative treatment, joint movement was encouraged on second postoperative day for every patient in both groups. The time to start weight-bearing differed widely. In our opinion, the appropriate time to begin with weight-bearing depends not only on the implant used, but also on the fracture type, postoperative stability, osteoporosis, and body weight as well. Haidukewych [20] highlighted 4 classic intertrochanteric fracture patterns that signify instability. The unstable patterns include reverse obliquity fractures, transtrochanteric fractures, fractures with a large posteromedial fragment implying loss of the calcar buttress, and fractures with subtrochanteric extension. He suggested nailing for these fractures. In this study, weight-bearing was delayed in patients with these classic fracture patterns, regardless of treatment groups.

A weakness of this study is that we are familiar with PFNA but not with reverse LISS, for it is originally designed for distal femur. Another weakness is the relatively small patient group. Further studies are required concerning LISS application to proximal femur. 

In conclusion, the results of the present study show that both the PFNA and the reverse LISS provide effective methods of treatment for intertrochanteric hip fractures. PFNA is superior to reverse LISS in terms of surgical time, weight-bearing, and perhaps fluoroscopy time. Mechanical failure can be minimized when the rehabilitation program is made based on individual characteristics.

## Figures and Tables

**Table 1:** Main demographic and clinical data of the fractures by treatment group.

	PFNA	Reverse LISS	*P* value
	(*n* = 10)	(*n* = 9)	
Type 31-A1 fractures (*n* = 19)			

Mean age (yr.)	80.1 ± 6.4	80.3 ± 8.1	0.945
Sex			
Male	6	2	0.096
Female	4	7	
Preoperative walking ability			
Independent walking	10	9	
Assisted walking	0	0	
Bedridden	0	0	
Mean duration from injury to surgery (day)	6.80 ± 3.3	6.11 ± 3.6	0.666
Mean duration of surgery (min.)	61.0 ± 9.4	87.2 ± 11.5	0.000
Mean fluoroscopy time (sec.)	109 ± 52.9	180 ± 70.8	0.024
Mean blood loss (mL)	210 ± 87.6	233 ± 82.9	0.560
Open reduction cases	0	0	
Quality of reduction			
Good	10	8	0.279
Acceptable	0	1	
Poor	0	0	
Mean time of hospital stay (day)	18.6 ± 3.1	21.3 ± 9.3	0.438
Mean time to bone healing (wk.)	17.4 ± 3.4	20.6 ± 3.2	0.054
Postoperative walking ability			
Independent walking	8	8	
Assisted walking	2	1	
Bedridden	0	0	
Harris hip score (pt.)	83.6 ± 5.8	85.2 ± 6.4	0.568

	PFNA	Reverse LISS	*P* value
	(*n* = 21)	(*n* = 21)	

Type 31-A2 fractures (*n* = 42)			

Mean age (yr.)	82.5 ± 7.9	80.7 ± 8.1	0.469
Sex			
Male	5	11	0.057
Female	16	10	
Preoperative walking ability			
Independent walking	17	19	
Assisted walking	3	2	
Bedridden	1	0	
Mean duration from injury to surgery (day)	6.57 ± 3.5	6.71 ± 4.8	0.912
Mean duration of surgery (min.)	65.5 ± 13.2	92.6 ± 17.1	0.000
Mean fluoroscopy time (sec.)	153 ± 59.7	202 ± 91.1	0.050
Mean blood loss (mL)	231 ± 100.6	248 ± 152.9	0.679
Open reduction cases	2	3	
Quality of reduction			
Good	19	20	0.549
Acceptable	2	1	
Poor	0	0	
Mean time of hospital stay (day)	19.5 ± 5.2	19.8 ± 6.0	0.847
Mean time to bone healing (wk.)	22.2 ± 3.6	23.1 ± 4.3	0.440
Postoperative walking ability			
Independent walking	17	14	
Assisted walking	3	6	
Bedridden	1	1	
Harris hip score (pt.)	81.4 ± 10.0	78.1 ± 12.6	0.353

	PFNA	Reverse LISS	*P* value
	(*n* = 14)	(*n* = 12)	

Type 31-A3 fractures (*n* = 26)			

Mean age (yr.)	77.4 ± 6.3	77.2 ± 6.4	0.917
Sex			
Male	5	4	0.899
Female	9	8	
Preoperative walking ability			
Independent walking	14	12	
Assisted walking	0	0	
Bedridden	0	0	
Mean duration from injury to surgery (day)	4.50 ± 2.1	5.17 ± 2.2	0.430
Mean duration of surgery (min.)	73.2 ± 15.4	97.5 ± 18.4	0.001
Mean fluoroscopy time (sec.)	142 ± 72.3	199 ± 88.9	0.084
Mean blood loss (mL)	236 ± 111.7	238 ± 98.0	0.966
Open reduction cases	1	1	
Quality of reduction			
Good	12	11	0.636
Acceptable	2	1	
Poor	0	0	
Mean time of hospital stay (day)	16.6 ± 1.95	20.2 ± 3.86	0.005
Mean time to bone healing (wk.)	22.0 ± 4.31	24.8 ± 3.07	0.070
Postoperative walking ability			
Independent walking	12	12	
Assisted walking	1	0	
Bedridden	1	0	
Harris hip score (pt.)	84.1 ± 11.3	86.2 ± 5.64	0.563

	PFNA	Reverse LISS	*P* value
	(*n* = 45)	(*n* = 42)	

All the fractures (*n* = 87)			

Mean age (yr.)	80.4 ± 7.3	79.6 ± 7.6	0.627
Sex			
Male	16	17	0.636
Female	29	25	
Preoperative walking ability			
Independent walking	41	40	
Assisted walking	3	2	
Bedridden	1	0	
Mean duration from injury to surgery (day)	5.98 ± 3.2	6.14 ± 3.9	0.828
Mean duration of surgery (min.)	66.9 ± 13.7	92.9 ± 16.5	0.000
Mean fluoroscopy time (sec.)	140 ± 63.5	196 ± 85.0	0.001
Mean blood loss (mL)	228 ± 100	242 ± 124	0.565
Open reduction cases	3	4	
Quality of reduction			
Good	41	40	0.765
Acceptable	4	2	
Poor	0	0	
Mean time of hospital stay (day)	18.4 ± 4.1	20.3 ± 6.3	0.101
Mean time to bone healing (wk.)	21.1 ± 4.2	23.1 ± 4.0	0.025
Postoperative walking ability			
Independent walking	37	34	
Assisted walking	6	7	
Bedridden	2	1	
Harris hip score (pt.)	82.8 ± 9.5	82.0 ± 10.4	0.717
Postoperative complications (cases)	4 (9%)	5 (12%)	
Pressure sore	1	2	
Urinary infection	1	2	
Pulmonary infection	1	1	
Deep venous thrombosis	1	0	
Death (cases)	4 (9%)	3 (7%)	
